# Preventing overuse of laboratory diagnostics: a case study into diagnosing anaemia in Dutch general practice

**DOI:** 10.1186/s12911-020-01198-8

**Published:** 2020-07-31

**Authors:** Michelle M. A. Kip, Martijn L. J. Oonk, Mark-David Levin, Annemarie Schop, Patrick J. E. Bindels, Ron Kusters, Hendrik Koffijberg

**Affiliations:** 1 Department of Health Technology and Services Research, University of Twente, Technical Medical Center, Faculty of Behavioural, Management and Social Sciences, Enschede, the Netherlands; 2 Department of Internal Medicine, Albert Schweitzer Hospital, Dordrecht, the Netherlands; 3 Department of General Practice, Erasmus MC, Rotterdam, the Netherlands; 4 Laboratory for Clinical Chemistry and Haematology, Jeroen Bosch Hospital, Den Bosch, the Netherlands

**Keywords:** Anemia, Data analysis, statistical, Diagnoses and laboratory examinations, General practice, Optimal testing, Overuse

## Abstract

**Background:**

More information is often thought to improve medical decision-making, which may lead to test overuse. This study assesses which out of 15 laboratory tests contribute to diagnosing the underlying cause of anaemia by general practitioners (GPs) and determines a potentially more efficient subset of tests for setting the correct diagnosis.

**Methods:**

Logistic regression was performed to determine the impact of individual tests on the (correct) diagnosis. The statistically optimal test subset for diagnosing a (correct) underlying cause of anaemia by GPs was determined using data from a previous survey including cases of real-world anaemia patients.

**Results:**

Only 9 (60%) of the laboratory tests, and patient age, contributed significantly to the GPs’ ability to diagnose an underlying cause of anaemia (CRP, ESR, ferritin, folic acid, haemoglobin, leukocytes, eGFR/MDRD, reticulocytes and serum iron). Diagnosing the correct underlying cause may require just five (33%) tests (CRP, ferritin, folic acid, MCV and transferrin), and patient age.

**Conclusions:**

In diagnosing the underlying cause of anaemia a subset of five tests has most added value. The real-world impact of using only this subset should be further investigated. As illustrated in this case study, a statistical approach to assessing the added value of tests may reduce test overuse.

## Background

In the last decades there has been a strong rise in the number of relatively cheap laboratory tests that are available as well as in the number of tests requested by physicians [1, 2]. These developments provide challenges to physicians with regard to determining which tests to order and how to interpret their combined results [3–5]. This issue is particularly relevant for general practitioners (GPs) as they order a large variety of laboratory tests during ~ 30% of all patient encounters [3, 4]. Although these challenges are partly unavoidable owing to the large variability of symptoms encountered within general practices [6], GPs may benefit from improved guidance as to which tests to order in which patients.

Besides the benefits of laboratory testing in terms of setting a diagnosis and deciding upon the best treatment strategy, these tests may offer wider benefits to patients, for example in terms of reducing diagnostic uncertainty or offering reassurance [6–8]. However, laboratory testing is (inevitably) also associated with patient discomfort, and a plethora of test results may divert the physician’s attention away from the clinically relevant information [9]. In addition, it may lead to overdiagnosis, which may result in unnecessary, potentially harmful or costly downstream activities [10–14]. Indeed, previous studies suggest that, depending on the definition used, 30–70% of all laboratory tests may be considered potentially inappropriate [6, 15, 16]. In this study, ‘overutilization’ of tests is defined as performing tests that do not affect medical decision-making [16, 17].

To investigate the added diagnostic value of tests for decision-making, a case study of patients presenting with anaemia in general practice is used. Anaemia is a frequently encountered condition in general practice and is characterized by a low blood haemoglobin level. Its incidence increases with age, and it is associated with increased morbidity and mortality [18–25]. However, as anaemia is not considered a disease in itself but rather a sign of a range of underlying conditions, the underlying cause is often under-diagnosed [26, 27]. Besides an anamnesis and physical examination, (a range of) laboratory tests are essential in the diagnostic work-up [28, 29]. The Dutch College of General Practitioners (DCGP)-guideline provides a flowchart supporting GPs in deciding which tests to order based on patient’s symptoms, their medical history, and the clinical suspicion [28]. The tests included in this flowchart include C-reactive protein (CRP), creatinine, erythrocyte sedimentation rate (ESR), ferritin, folic acid, haemoglobin, lactate dehydrogenase, leukocytes, mean corpuscular volume (MCV), reticulocytes, serum iron, thrombocytes, transferrin and vitamin B12. Besides these 14 tests, the patient’s renal function (i.e. the eGFR [or MDRD]) is calculated, serving as 15th test result.

Despite following the DCGP-guideline, the underlying cause of anaemia remains unknown in 52% of patients [30]. Previous research indicated that immediately ordering all 15 tests in anaemia patients improves GPs’ ability to correctly diagnose the underlying cause and is cost-effective compared to letting GPs decide themselves which tests to order [31, 32]. It is however unknown whether all 15 tests individually contribute to the GP’s ability to (correctly) diagnose the underlying cause of anaemia. Therefore, the current study investigates to what extent each individual test (*within* the full set of 15 tests) adds value to this diagnostic process. In addition, the optimal subset of relevant tests will be determined from a statistical perspective.

## Methods

### Study design

The data used for this analysis were obtained from a previously conducted questionnaire, in which GPs were asked to determine the underlying cause in cases of real-world anaemia patients. In this section, an explanation of the questionnaire and the database used in this questionnaire will be provided. A more extensive description was published previously [31].

The cases used in this questionnaire were obtained from a prospective database including patients aged ≥50 years presenting with newly diagnosed anaemia in general practice (*n* = 2389). This database excluded patients with multiple underlying causes (*n* = 293) and contained information about patients’ age, gender, and the results of all abovementioned 15 tests. From this database, 201 cases were randomly selected to be used in the questionnaire. In this random selection, the actual prevalence of each of the underlying causes of anaemia was maintained [32]. The characteristics of the 201 cases used in this questionnaire are presented in Table 1. In the questionnaire, GPs (*n* = 139) were presented with cases of anaemia patients. For each GP, six cases of anaemia patients were randomly drawn from the set of 201 cases. For each of these six cases, the GP was asked to establish the underlying cause based on the patient’s age, gender, and test results. In three out of these six cases, GPs were immediately provided with all 15 test results. In the other three cases, GPs were asked to decide for themselves which tests to perform. These questions were however excluded from the current analysis as this analysis only focused on the three cases in which the GP received the full set of 15 test results. The underlying causes to choose from were anaemia of chronic disease (ACD), iron deficiency anaemia (IDA) and renal anaemia (RA). In addition, GPs could choose the option ‘other’ in which they were asked to specify the expected underlying cause of anaemia, or they could indicate that they could not establish the underlying cause based on the information provided (classified as ‘unknown’). For each case, the correctness of this underlying cause was determined by comparing it with its (presumably) correct diagnosis as established by an expert panel, consisting of a GP, an internist and a clinical chemist [31].

**Table 1:** Descriptive statistics. Descriptive statistics of test results

	Mean	SD	Range	Normal, % (n)	Abnormal, % (n)
**Tests with numerical results: mean, SD, and frequency of result within reference value**^**a**^**(*n*** **= 201 cases)**					
ESR (mm/h)	34.7	27.3	0.0–120.0	60% (120)	40% (81)
CRP (mg/L)	26.5	46.9	4.0–290.0	67% (135)	33% (66)
Haemoglobin (mmol/L)	7.3	0.8	4.2–8.4	0% (0)	100% (201)
Reticulocytes (% of RBCs)	1.0	0.5	0.3–4.8	98% (197)	2% (4)
Creatinine (μmol/L)	91.5	49.4	42.0–449.0	67% (135)	Low: 3% [6];
					High: 30% (60)
eGFR (mL/min/1,73m^2^)	71.0	25.8	8.0–184.0	62% (124)	38% (77)
LDH (E/L)	399.5	652.7	126.0–9385.0	84% (169)	16% (32)
Serum iron (μmol/L)	10.1	5.3	1.9–25.4	36% (73)	64% (128)
Folic acid (nmol/L)	21.6	20.1	3.0–227.0	98% (197)	2% (4)
Vitamin B12 (pmol/L)	335.1	182.6	102.0–1408.0	97% (195)	3% (6)
**Tests with categorical results: frequency of result within reference value**^**a**^**(n = 201 cases)**					
Ferritin (μg/L)	196.6	316.5	2.0–3322.0	Low normal: 35% (70) High normal: 24% (49)	Low: 13% (25)
					High: 29% (57)
Leukocytes (× 10^9^/L)	7.7	3.1	2.0–25.3	78% (157)	Low: 6% (13)
					High 15% (31)
Thrombocytes (× 10^9^/L)	295.5	107.9	117.0–782.0	82% (165)	Low: 2% (5)
					High: 15% (31)
MCV (fL)	90.0	8.3	60.0–130.0	84% (168)	Low: 9% (18)
					High: 8% (15)
Transferrin (g/L)	2.5	0.6	1.0–4.1	76% (152)	Low: 18% (36)
					High: 6% (13)

*CRP* C-reactive protein, *eGFR* estimated glomerular filtration rate, *ESR* erythrocyte sedimentation rate, *LDH* lactate dehydrogenase, *MCV* mean corpuscular volume, *RBCs* red blood cells, *SD* standard deviation

^a^Reference values applied in the Albert Schweitzer Hospital were used to determine whether a test result was normal or abnormal

**Fig. 1:**
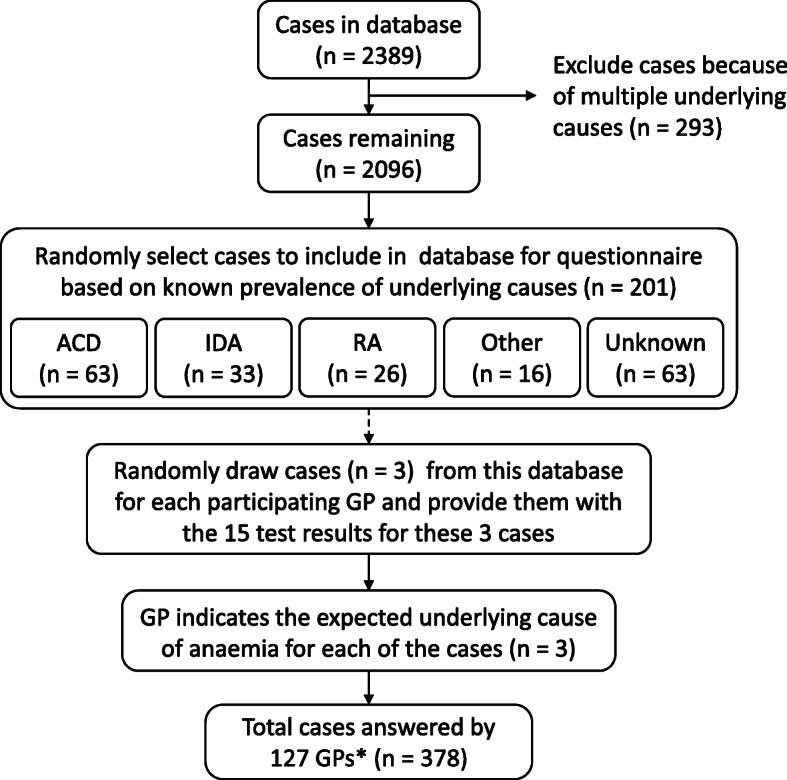
Overview of case selection and cases presented in questionnaire. This figure presents an overview of how the cases were selected from the database and presented to the GPs who participated in the questionnaire. *125 GPs completed all 3 cases, 1 GP completed only 2 cases and 1 GP completed only 1 case. ACD = anaemia of chronic disease, GP = general practitioner, IDA = iron deficiency anaemia, RA = renal anaemia

Altogether, the questionnaire resulted in 378 anaemia cases in whom an underlying cause was diagnosed by a GP based on the full set of 15 test results. An overview of the entire process of case selection and presenting these to the GPs is shown in Fig. 1.

### Data analysis

The data obtained from the 378 cases, including the results of the 15 test for each case, the underlying cause of anaemia as indicated by the GP, as well as the correct underlying cause according to the expert panel, were used as input for the current analysis. Data were analyzed using R (version 3.5.0) [33]. The package mice (version 3.30) was used for single regression imputation of missing data for the ESR test (*n* = 20) [34].

As it was considered unlikely that other, less common, causes of anaemia can be diagnosed in general practice (based on the limited information provided) [31], this study specifically focused on correctly diagnosing IDA, ACD and RA. Therefore, the diagnoses ‘other’ and ‘unknown’ were considered as one category. For CRP and vitamin B12, the non-numerical values “smaller than 5 mg/L” (*n* = 109) and “smaller than 111 pmol/L” (*n* = 2) were replaced by the numerical values 4 mg/L and 110 pmol/L, respectively.

For patients with anaemia, the majority of the 15 tests analyzed in this study have one single cut-off value to indicate abnormal test results, for example a haemoglobin level < 8.5 mmol/L (i.e. < 13.7 g/dL) in male patients. These test results were therefore handled as binary variables (i.e. normal/abnormal). However, five tests (i.e. ferritin, leukocytes, MCV, thrombocytes and transferrin) can have a too low as well as a too high test result. As too low and too high values are often indicative of different underlying causes of anaemia, the numerical values of these five tests were converted to categorical results. A detailed overview of the cut-off values used (based on the DCGP-guideline [28]) is provided in Table S1 of Additional file 1.

#### The value of individual tests for (correctly) diagnosing an underlying cause of anaemia

The impact on the GPs’ ability to diagnose an underlying cause of anaemia was investigated in two ways: 1) for diagnosing *an* underlying cause of anaemia (regardless whether this diagnosis was correct), 2) for diagnosing the *correct* underlying cause of anaemia. For investigating the impact of an individual test result on GPs’ ability to diagnose *an* underlying cause, the impact of each of the 15 tests (within this *complete* set of tests) was assessed separately by fitting a multinomial logistic regression model (MLR) with a logistic link function, using the mlogit-package and nnet-package [35, 36]. In addition to the 15 test results, the patients’ age and gender were also considered as predictors in the fitting process. The MLR shows the impact of a one unit change in the predictor (for example a change in ESR from 35 to 36 mm/h) on the log odds of the GP diagnosing a specific cause of anaemia rather than diagnosing ‘unknown’. The goal of this analysis was to assess whether specific test outcomes may substantially affect the likelihood of diagnosing *one* specific underlying cause of anaemia, while not affecting the diagnosis of any of the other underlying causes. In other words, it is determined whether the GPs incorporate this test in their decision to diagnose a specific underlying cause of anaemia.

Subsequently, the added value of each test (within the *complete* set of tests) on the GP’s ability to diagnose the *correct* underlying cause was assessed by fitting a binomial logistic regression model (BLR). In contrast to the MLR, the BLR shows the impact of a one unit change in test result on the log odds of the GP diagnosing the *correct* rather than an *incorrect* underlying cause (for details see Additional file 1).

#### The combined value of tests for (correctly) diagnosing an underlying cause of anaemia

**Fig. 2:**
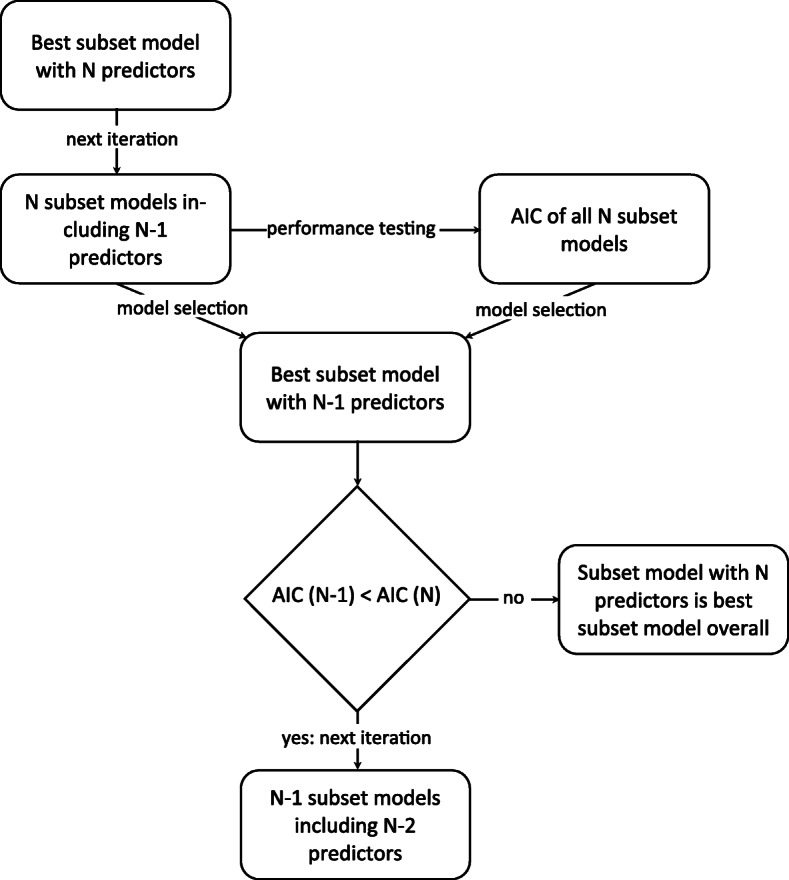
Overview of the best subset selection process. This figure illustrates the steps taken in selecting the best (i.e. statistically most efficient) subset of laboratory tests. AIC = Akaike Information Criterion

In current practice, many of the 15 tests are ordered simultaneously [31]. However, an overlap may exist between the information they provide and hence their impact on the GP’s ability to (correctly) diagnose the underlying cause. Consequently, a *subset* of tests may actually suffice. Yet, this overlap cannot be captured using a single MLR or BLR model, because the added value of a test may depend on the availability of other test results. Therefore, this overlap was determined, and (largely) removed, by identifying the statistically most efficient test subset (i.e. best subset) using stepwise backward selection [37]. This analysis was performed twice: with and without considering the correctness of the diagnosed underlying cause. The Akaike Information Criterion (AIC) was used as performance indicator to balance model complexity (i.e. number of predictors) and goodness-of-fit of the model [38]. In this process, the initial set of 17 predictors (i.e. age, gender and 15 test results) was iteratively reduced by removing the predictor with the lowest impact on the ability of the GP to (correctly) diagnose the underlying cause of anaemia. During each iteration, the AIC was determined, with the subset model with the lowest AIC being the most favorable. This process was repeated until the AIC could not be decreased further (Fig. 2), indicating that reducing model complexity by removing one more predictor resulted in substantially worse performance.

#### Testing assumptions

Finally, several assumptions underlying the logistic regression model were tested, including testing for multicollinearity, perfect separation of predictors, and a Hausman-McFadden test (for details see Additional file 1).

This research did not receive any specific grant from funding agencies in the public, commercial, or not-for-profit sectors.

## Results

As mentioned previously, the questionnaire resulted in 378 anaemia cases that were included in the current analysis. Of these cases, 117 (31.0%) were diagnosed as ACD, 76 (20.1%) as IDA, 50 (13.2%) as RA, 22 (5.8%) as ‘other’ and 113 (29.9%) as ‘unknown’. Comparing these diagnoses with the diagnoses by the expert panel indicated that 234 (61.9%) were correct, including 47 (61.8%) of the IDA diagnoses, 73 (62.4%) of the ACD diagnoses, 29 (58.0%) of the RA diagnoses, 17 (77.3%) of the ‘other underlying causes’ and 68 (60.2%) of the ‘unknown’.

These 378 cases were based on 201 cases of anaemia patients, indicating that some cases were answered multiple times (by different GPs). Of these 201 patients, 94 (47%) were male, 107 (53%) were female, and the mean age was 74.7 years (range: 50–102 years).

### The value of individual tests for (correctly) diagnosing an underlying cause of anaemia

When considering the impact of *individual* test results within the complete set of tests, the MLR indicates that CRP, ESR, ferritin, folic acid, leukocytes, eGFR, reticulocytes and serum iron significantly impact the ability of the GP to diagnose *an* underlying cause of anaemia (instead of ‘unknown’), regardless whether this diagnosis is correct, as shown in Table 2. Each row in Table 2 indicates the effect of a change in the test result on the probability that the GP diagnoses a *specific* underlying cause, rather than diagnosing an ‘unknown’ cause. For the tests with numerical results (i.e. ESR, eGFR, folic acid, reticulocytes and serum iron), this ‘change’ implies a *one unit* shift in the test result, for example a shift in ESR from 36 to 35 mm/h. For the categorized test results, the table mentions the specific shift in the result category (i.e. from normal to abnormal for leukocytes, and a shift from low normal to either low, high normal, or to high, for ferritin). The results of the other seven tests (i.e. creatinine, CRP, haemoglobin, LDH, MCV, thrombocytes, transferrin and vitamin B12) do not significantly contribute to the ability of the GP to diagnose *an* underlying cause. In other words, the extent to which the GP considers this test result in diagnosing an underlying cause of anaemia is insufficient to have a statistically significant impact. As this analysis involves many different comparisons, only the significant outcomes are shown in this table.

**Table 2:** Impact of laboratory tests on diagnose an underlying cause of anaemia by GPs. This table shows the impact of individual test results (within the complete set of tests) on the ability of the GP to diagnose an underlying cause of anaemia, regardless whether this diagnosis is correct

Tests with numerical results					
Test		Underlying cause instead of unknown	Coefficient (log odds^*a*^)	Exponentiated coefficient (odds)	*p*-value
ESR		ACD	0.031	1.032	< 0.001***
ESR		RA	−0.044	0.957	0.047*
ESR		Other	−0.060	0.941	0.022*
CRP		RA	−0.065	0.937	0.043*
Reticulocytes		Other	3.650	38.471	< 0.001***
eGFR		RA	−0.250	0.779	< 0.001***
Folic acid		Other	−0.091	0.913	0.023*
Serum iron		IDA	−0.158	0.854	0.024*
Tests with categorical results					
Test	Shift in the result category	Underlying cause instead of unknown	Coefficient (log odds^*a*^)	Exponentiated coefficient (odds)	p-value
Leukocytes	Normal to abnormal	Other	2.352	10.503	0.005**
Ferritin	Low normal to low	IDA	3.049	21.088	< 0.001***
Ferritin	Low normal to high normal	ACD	1.310	3.706	0.003**
Ferritin	Low normal to high normal	IDA	1.364	3.911	0.031*
Ferritin	Low normal to high	ACD	1.026	2.791	0.014*
Ferritin	Low normal to high	IDA	1.469	4.343	0.029*
Ferritin	Low normal to high	Other	−4.690	0.009	0.002**

*ACD* anaemia of chronic disease, *CRP* C-reactive protein, *eGFR* estimated glomerular filtration rate, *ESR* erythrocyte sedimentation rate, *IDA* iron deficiency anaemia, *RA* renal anaemia

Significance levels: *** = 0.001; ** = 0.01; * = 0.05

^a^For a more detailed explanation on the interpretation of the values reported in this table, see Additional file 1

Table 3 shows the results of the BLR, indicating the impact of a change in the result of the *individual* tests, within the complete set of tests, on the ability of the GP to diagnose the *correct* underlying cause of anaemia. Results indicate a statistically significant impact for a shift from a normal to a high MCV, as well as a shift from a low normal to a high normal ferritin level, and from a low normal to low ferritin level. For the other tests with categorical results (i.e. leukocytes, thrombocytes, and transferrin), as well as all tests with numerical results (i.e. creatinine, CRP, eGFR, ESR, folic acid, haemoglobin, LDH, serum iron and vitamin B12) no significant impact of a one unit change in a *single* test result on the ability of the GP to *correctly* diagnose the underlying cause of anaemia was found.

**Table 3:** Impact of laboratory tests on diagnosing the correct underlying cause of anaemia by GPs. This table shows the impact of individual test results (within the complete set of tests) on the ability of the GP to diagnose the correct underlying cause of anaemia

Tests with numerical results				
Test		Coefficient (log odds^a^)	Exponentiated coefficient (odds)	*p*-value
ESR		−0.004	0.996	0.578
CRP		0.008	1.008	0.070
Haemoglobin		−0.216	0.806	0.395
Reticulocytes		0.176	0.192	0.560
Creatinine		0.006	1.006	0.222
eGFR		0.015	1.015	0.094
LDH		0.000	1.001	0.641
Serum iron		0.024	1.024	0.482
Folic acid		0.013	1.014	0.075
Vitamin B12		−0.000	1.000	0.509
Tests with categorical results				
Test	Shift in the result category	Coefficient (log odds^a^)	Exponentiated coefficient (odds)	p-value
MCV	Normal to high	1.600	4.954	0.006 **
MCV	Normal to low	−0.176	0.838	0.785
Ferritin	Low normal to high	−0.123	0.884	0.725
Ferritin	Low normal to high normal	−0.634	0.531	0.047 *
Ferritin	Low normal to low	1.231	3.425	0.030 *
Leukocytes	Normal to abnormal	−0.169	0.844	0.584
Thrombocytes	Normal to abnormal	0.126	1.134	0.730
Transferrin	Normal to high	0.246	1.279	0.741
Transferrin	Normal to low	−0.781	0.458	0.051

*CRP* C-reactive protein, *eGFR* estimated glomerular filtration rate, *ESR* erythrocyte sedimentation rate, *LDH* lactate dehydrogenase, *MCV* mean corpuscular volume

Significance levels: *** = 0.001; ** = 0.01; * = 0.05

^a^For a more detailed explanation on the interpretation of the values reported in this table, see Additional file 1

### The combined value of tests for (correctly) diagnosing an underlying cause of anaemia

When considering the value of a combination of test results, the most efficient test subset for diagnosing *an* underlying cause as well as for diagnosing the *correct* underlying cause are shown (Table 4). For diagnosing *an* underlying cause, seven predictors were eliminated from the initial set of 17 predictors (i.e. age, gender and the 15 test results). The 10 remaining predictors include the patient characteristic ‘age’, and the tests CRP, ESR, ferritin, folic acid, haemoglobin, leukocytes, eGFR, reticulocytes and serum iron. For diagnosing the *correct* underlying cause, 11 predictors were eliminated. The six remaining predictors include the patient characteristic ‘age’ and the tests CRP, ferritin, folic acid, MCV and transferrin. For details see Tables S2 and S3 of Additional file 1.

**Table 4:** Result of best subset selection. This table shows the result of best subset (i.e. most efficient subset) selection for the two patient characteristics and the 15 test results, for diagnosing an underlying cause as well as for diagnosing the correct underlying cause

	Predictors included after best subset selection		
	Predictors	For diagnosing an underlying cause	For diagnosing the correct underlying cause
Patient characteristics	Age	X	X
	Gender		
Test results	Creatinine		
	CRP	X	X
	ESR	X	
	Ferritin	X	X
	Folic acid	X	X
	Haemoglobin	X	
	LDH		
	Leukocytes	X	
	MCV		X
	eGFR	X	
	Reticulocytes	X	
	Serum iron	X	
	Thrombocytes		
	Transferrin		X
	Vitamin B12		

*CRP* C-reactive protein, *eGFR* estimated glomerular filtration rate, *ESR* erythrocyte sedimentation rate, *LDH* lactate dehydrogenase, *MCV* mean corpuscular volume

### Testing assumptions

All variance inflation factors determined were < 5 (Table S4 of Additional file 1), all maximum likelihood estimators converged, and the independence of irrelevant alternatives assumption was not rejected.

## Discussion

Of the 15 tests evaluated, only a subset impacts the GP’s ability to (correctly) diagnose an underlying cause of anaemia, from a statistical perspective. The statistically most efficient subset of predictors for diagnosing the *correct* underlying cause contains, besides a patient’s age, five tests: ferritin, CRP, MCV, transferrin and folic acid.

However, when considering predictors for diagnosing *an* underlying cause of anaemia, the statistically most efficient subset contains, besides a patient’s age, nine tests (i.e. CRP, ESR, ferritin, folic acid, haemoglobin, leukocytes, eGFR, reticulocytes and serum iron). For some of these tests, a strong relation may exist between the test outcome and the probability that the GP diagnoses a specific underlying cause, regardless of whether this underlying cause is correct. In other words, when tests contribute to diagnosing *an* underlying cause by the GP (as shown in Table 2), it implies that these tests are taken into account by the GP in their decision making process. However, this does not imply that the test also has a (statistically significant) impact on diagnosing the *correct* underlying cause (as shown in Table 3). For example, a high ESR is often, but not always, caused by ACD [28]. Therefore, an elevated ESR test result might lead (too often) to this particular diagnosis, also if it is incorrect.

As shown in Table 4, the most efficient subset for diagnosing *an* underlying cause of anaemia includes the haemoglobin test, whereas the most efficient subset for diagnosing a *correct* underlying cause does not. This may be explained by the fact that a low haemoglobin level is a prerequisite for diagnosing anaemia without providing evidence on the underlying cause.

When considering the results of the BLR for *individual* tests, it is observed that only two of the statistically significant tests (i.e. ferritin and MCV) were found to also be part of the statistically most efficient *subset*, that is combination of tests, for diagnosing the correct underlying cause of anaemia. The other three tests in this subset (i.e. CRP, transferrin and folic acid) were not statistically significant when considered individually. This is most likely explained by the fact that these tests, in *combination*, are highly important for the GP to diagnose the correct underlying cause.

Besides the cases in whom the GPs and/or the expert panel were able to (correctly) diagnose an underlying cause of anaemia, an underlying cause could not be established in 113 out of 378 (i.e. 29.9%) of the cases by the GPs, and 63 out of 201 cases (i.e. 31.3%) by the expert panel, which is in line with literature [21, 39–41]. Further details on the suggested clinical management by the GPs (including medication prescriptions, referral to secondary care, etc.) was published previously [32].

### Strengths

As the analyses in this study are based on real-life patient data (in which the incidence of the underlying causes of anaemia mimics current practice) and because a representative sample of GPs responded to the survey [31, 32], the results likely provide a good representation of current practice. In addition, the incorporation of a patient’s age and gender as predictors further increases the reliability of the results, as these characteristics should be considered in a patient’s diagnostic work-up [28].

### Limitations

This study has certain limitations. First, although the GPs were provided with the patients’ age and gender, they should ideally also have been able to incorporate information about a patient’s anamnesis, medical history, physical examination, or the results of other diagnostic tests, in their diagnostic process [28]. For example, aspects like a history of renal failure may be highly important for diagnosing RA. However, as such aspects are (partly) dependent on the GP’s perception and experience, these cannot be comprehensively captured in a database. As both the GPs and the expert panel established the underlying cause of anaemia based on the (limited) information presented in the questionnaire and by using the available guidelines [28], this lack of information was similar in both groups. As a consequence, the underlying causes diagnosed in the questionnaire may not fully resemble the diagnosis that would have been established in clinical practice, but this effect is likely similar for GPs and the expert panel. It is therefore expected that this limitation did not affect the added value of the 15 tests as reported in this study. In addition, as the expert panel (i.e. a GP, an internist and a clinical chemist) established the underlying cause based on their broad expertise across these three disciplines, and because they used the current clinical guidelines, it was assumed that this diagnosis was correct or at least the best diagnosis that could be established based on the information provided.

Second, the analysis only considers the impact of laboratory tests on the ability of GPs to (correctly) diagnose the underlying cause of anaemia, and does not account for other aspects of the value of testing (e.g. in terms of patient reassurance). Third, the impact of individual tests on setting *a* diagnosis or a *correct* diagnosis is expressed in terms of a one unit change in the result of a single test. The expected variability in test results should therefore be considered in relation to its unit of measurement. For example, the reticulocyte test result usually varies between 1 and 2% [42], indicating that a one *unit* (i.e. 1%) change leads to a very high regression coefficient compared to a one *unit* (i.e. 1 mm/h) change in ESR. The difference in the impact of these changes in clinical practice is however likely less pronounced. In addition, in the current analysis, all numerical test results were classified as either normal or abnormal. Consequently, the analysis could not account for the potential impact of test results that deviate strongly from their reference value, compared with test results that only show a minor deviation, on the (correctness) of the diagnosed underlying cause. Finally, tests were evaluated and selected based on their overall contribution to the GP’s ability to correctly diagnose the underlying cause of anaemia. Consequently, tests that may only be valuable for diagnosing a specific (less common) cause may not be identified as such in the current analysis, where added value is essentially assessed across all patients and all underlying causes. This may (for example) explain why an abnormal creatinine test result was not significantly associated with (correctly) diagnosing RA, as only 26 out of 201 cases (12.9%) involved RA patients.

### Implications for practice

Annually, in the Netherlands, 57,000 patients are newly diagnosed with anaemia in general practice [31]. Compared to immediately ordering the full set of 15 tests, test overuse may be reduced with 67% while the percentage of correct diagnoses is expected to be (almost) unaffected. In practice in the Netherlands, GPs may (initially) order any number of tests, and on average they order seven tests [31]. Conversely, ordering just the five tests of the statistically most efficient subset, and ordering additional tests only when no clear underlying cause can be found based on this, could save up to two tests per patient (i.e. -29%) [32], while the percentage of patients with a correct diagnosis is expected to increase. Although cost savings in terms of preventing (unnecessary) laboratory tests are relatively small, the most efficient test subset may prevent unnecessary downstream diagnostic activities, thereby preventing unnecessary patient burden and reducing healthcare costs. However, decisions regarding which tests to perform in (suspected) anaemia patients are increasingly supported by clinical chemistry laboratories [43]. As the protocols for this ‘reflex-testing’ differ between hospitals, the results of this study are likely also valuable for laboratories to establish a standardized, optimal subset of laboratory tests for reflex-testing. As the work-up of establishing the underlying cause in newly diagnosed anaemia patients differs (slightly) between countries, this may limit the generalizability of the results presented here. The concept of overuse of laboratory tests is, however, not limited to the Netherlands and also not limited to anaemia patients. This study can therefore be considered an example of how statistical analyses can contribute to defining a potentially more efficient subset of laboratory tests and thereby to prevent test overuse. It is therefore recommended to perform similar studies in other countries, disease areas or medical conditions, in order to safely decrease the number of tests performed.

## Conclusions

Although current clinical guidelines recommend the use of an extensive set of laboratory tests to diagnose the underlying cause of anaemia, a subset of five tests has most added value from a statistical perspective. This subsets still provides a similar ability to the GP to (correctly) diagnose an underlying cause of anaemia. Consequently, a statistical approach to assessing the added value of tests may reduce test overuse. Whether such a subset of tests is acceptable and cost-effective in daily practice should be further investigated.

## Supplementary information

**Additional file 1.** This file contains the reference values of the 15 laboratory tests, extensive descriptions of the multinomial and the binomial logistic regression model, the assumptions that have been tested, as well as the detailed results of the multinomial logistic regression model, of the best subset selection and of the variance inflation factor.

## Data Availability

The data that support the findings of this study are available from the corresponding author, upon reasonable request.

## Abbreviations

ACD: anaemia of chronic disease
BLR: binomial logistic regression
CRP: C-reactive protein
DCGP: Dutch college of general practitioners
eGFR: estimated glomerular filtration rate
ESR: erythrocyte sedimentation rate
GP: general practitioner
IDA: iron deficiency anaemia
MCV: mean corpuscular volume
MDRD: modification of diet in renal disease
MLR: multinomial logistic regression
RA: renal anaemia

## References

[1] O'Sullivan JW, Stevens S, Hobbs FDR, Salisbury C, Little P, Goldacre B (2018). Temporal trends in use of tests in UK primary care, 2000-15: retrospective analysis of 250 million tests. BMJ..

[2] Jackson BR (2007). Managing laboratory test use: principles and tools. Clin Lab Med.

[3] Hickner J, Thompson PJ, Wilkinson T, Epner P, Sheehan M, Pollock AM (2014). Primary care physicians' challenges in ordering clinical laboratory tests and interpreting results. J Am Board of Family Med.

[4] Cadogan SL, Browne JP, Bradley CP, Cahill MR (2015). The effectiveness of interventions to improve laboratory requesting patterns among primary care physicians: a systematic review. Implementation Sci.

[5] Laposata M (2014). Putting the patient first--using the expertise of laboratory professionals to produce rapid and accurate diagnoses. Lab Med.

[6] van der Weijden T, van Bokhoven MA, Dinant GJ, van Hasselt CM, Grol RP (2002). Understanding laboratory testing in diagnostic uncertainty: a qualitative study in general practice. Brit J General Practice.

[7] Guthrie B (2009). Why do general practitioners take blood? A cross-sectional study of use of blood tests in UK general practice. Eur J General Practice.

[8] Bossuyt PM, Reitsma JB, Linnet K, Moons KG (2012). Beyond diagnostic accuracy: the clinical utility of diagnostic tests. Clin Chem.

[9] Elnenaei MO, Campbell SG, Thoni AJ, Lou A, Crocker BD, Nassar BA (2016). An effective utilization management strategy by dual approach of influencing physician ordering and gate keeping. Clin Biochem.

[10] Hall SF, Webber C, Groome PA, Booth CM, Nguyen P, DeWit Y (2019). Do doctors who order more routine medical tests diagnose more cancers? A population-based study from Ontario Canada. Cancer Med.

[11] Sohlberg EM, Metzner TJ, Leppert JT. The harms of Overdiagnosis and overtreatment in patients with small renal masses: a mini-review. Eur Urol Focus. 2019;5(6):943–5. 10.1016/j.euf.2019.03.006 30905599

[12] Webber BJ, Burganowski RP, Colton L, Escobar JD, Pathak SR, Gambino-Shirley KJ. Lyme disease overdiagnosis in a large healthcare system: a population-based, retrospective study. Clin Microbiol Infection. 2019;25(10):1233–8. 10.1016/j.cmi.2019.02.020 30802651

[13] Salerno S, Laghi A, Cantone MC, Sartori P, Pinto A, Frija G. Overdiagnosis and overimaging: an ethical issue for radiological protection. Radiol Med. 2019:124(8):714–20. 10.1007/s11547-019-01029-5 30900132

[14] Vickers AJ (2019). Redesigning prostate Cancer screening strategies to reduce Overdiagnosis. Clin Chem.

[15] Zhi M, Ding EL, Theisen-Toupal J, Whelan J, Arnaout R (2013). The landscape of inappropriate laboratory testing: a 15-year meta-analysis. PLoS One.

[16] Cadamuro J, Gaksch M, Wiedemann H, Lippi G, von Meyer A, Pertersmann A (2018). Are laboratory tests always needed? Frequency and causes of laboratory overuse in a hospital setting. Clin Biochem.

[17] Moons KG, de Groot JA, Linnet K, Reitsma JB, Bossuyt PM (2012). Quantifying the added value of a diagnostic test or marker. Clin Chem.

[18] Riva E, Tettamanti M, Mosconi P, Apolone G, Gandini F, Nobili A (2009). Association of mild anemia with hospitalization and mortality in the elderly: the health and Anemia population-based study. Haematologica..

[19] Lucca U, Tettamanti M, Mosconi P, Apolone G, Gandini F, Nobili A (2008). Association of mild anemia with cognitive, functional, mood and quality of life outcomes in the elderly: the "health and Anemia" study. PLoS One.

[20] Smith RE (2010). The clinical and economic burden of anemia. Am J Managed Care.

[21] Shavelle RM, MacKenzie R, Paculdo DR (2012). Anemia and mortality in older persons: does the type of anemia affect survival?. Int J Hematol.

[22] Zakai NA, Katz R, Hirsch C, Shlipak MG, Chaves PH, Newman AB (2005). A prospective study of anemia status, hemoglobin concentration, and mortality in an elderly cohort: the cardiovascular health study. Arch Intern Med.

[23] Steensma DP, Tefferi A (2007). Anemia in the elderly: how should we define it, when does it matter, and what can be done?. Mayo Clin Proc.

[24] Culleton BF, Manns BJ, Zhang J, Tonelli M, Klarenbach S, Hemmelgarn BR (2006). Impact of anemia on hospitalization and mortality in older adults. Blood..

[25] Penninx BW, Pahor M, Woodman RC, Guralnik JM (2006). Anemia in old age is associated with increased mortality and hospitalization. J Gerontol A Biol Sci Med Sci.

[26] Thomas DR (2004). Anemia and quality of life: unrecognized and undertreated. J Gerontol A Biol Sci Med Sci.

[27] Nissenson AR, Goodnough LT, Dubois RW (2003). Anemia: not just an innocent bystander?. Arch Intern Med.

[28] Van Wijk MAM, Mel M, Muller PA, et al. Nederlands Huisartsen Genootschap – Standaard Anemie (Revisie). Huisarts Wet. 2014;57(10):528–36.

[29] Smith A. Guide to evaluation and treatment of anaemia in general practice. Drug Review Anaemia. 2012:25–42.

[30] Oosterhuis WP, Van der Horst M, van Dongen K, Ulenkate HJLM, Volmer M, Wulkan RW (2007). Prospective comparison of the flow chart for laboratory investigations for anaemia from the Dutch College of General Practitioners' guideline 'Anaemia' with a self-developed, substantive and logistical alternative flow chart'. Ned Tijdschr Geneeskd.

[31] Schop A, Kip MM, Stouten K, Dekker S, Riedl J, van Houten RJ (2018). The effectiveness of a routine versus an extensive laboratory analysis in the diagnosis of anaemia in general practice. Ann Clin Biochem.

[32] Kip MM, Schop A, Stouten K, Dekker S, Dinant GJ, Koffijberg H (2018). Assessing the cost-effectiveness of a routine versus an extensive laboratory work-up in the diagnosis of anaemia in Dutch general practice. Ann Clin Biochem.

[33] R Core Team (2019). R: a languange and environment for statistical computing.

[34] van Buuren S, Groothuis-Oudshoorn K (2011). Mice: multivariate imputation by chained equations in R. J Stat Softw.

[35] Croissant Y. mlogit: Multinomial Logit Models. R package version 1.1–0. https://CRAN.R-project.org/package=mlogit. 2020.

[36] Venables WNR, B. D. Modern Applied Statistics with S. Fourth Edition. Springer, New York. ISBN 0–387–95457-0. 2002.

[37] James G (2014). An introduction to statistical learning: with applications in R.

[38] Chaurasia A, Harel O (2012). Using AIC in multiple linear regression framework with multiply imputed data. Health Serv Outcome Res Methodol.

[39] Eisele L, Durig J, Broecker-Preuss M, Duhrsen U, Bokhof B, Erbel R (2013). Prevalence and incidence of anemia in the German Heinz Nixdorf recall study. Ann Hematol.

[40] Patel KV (2008). Epidemiology of anemia in older adults. Semin Hematol.

[41] Ferrucci L, Semba RD, Guralnik JM, Ershler WB, Bandinelli S, Patel KV (2010). Proinflammatory state, hepcidin, and anemia in older persons. Blood..

[42] Stehouwer CDA. Interne Geneeskunde. 2010:248.

[43] Verboeket - van de Venne WPHG, Oosterhuis WP, Kleinveld HA, Leers MPG. Anemieprotocollen voor de eerste lijn in Nederland. 63e Congres van de Nederlandse Vereniging voor Klinische Chemie en Laboratoriumgeneeskunde; Veldhoven: Ned Tijdschr Klin Chem Labgeneesk; 2010. p. 91–133.

