# Supplementary material for: Preventing overuse of laboratory diagnostics: a case study into diagnosing anaemia in Dutch general practice
Source: BMC Med Inform Decis Mak. 2020 Jul 31;20:178. doi: 10.1186/s12911-020-01198-8 (PMC7395377; doi:10.1186/s12911-020-01198-8)
Supplement: Supplementary file 1 — Additional file 1. This file contains the reference values of the 15 laboratory tests, extensive descriptions of the multinomial and the binomial logistic regression model, the assumptions that have been tested, as well as the detailed results of the multinomial logistic regression model, of the best subset selection and of the variance inflation factor. [file 12911_2020_1198_MOESM1_ESM.doc]

**Additional file 1**

1. **Reference values of the 15 laboratory tests**

**Table S1**. Overview of reference values used for each of the 15 laboratory tests.

| Tests with numerical results | | | | | | |
| --- | --- | --- | --- | --- | --- | --- |
| ESR* | abnormal | | | normal | | |
| Male <70 years | >25 mm/h | | | ≤25 mm/h | | |
| Male ≥70 years | >35 mm/h | | | ≤35 mm/h | | |
| Female <70 years | >30 mm/h | | | ≤30 mm/h | | |
| Female ≥70 years | >35 mm/h | | | ≤35 mm/h | | |
| CRP | abnormal | | | normal | | |
| All cases | >10 mg/L | | | ≤10 mg/L | | |
| Haemoglobin | abnormal | | | normal | | |
| Male | <13.7 g/dL (i.e. <8.5 mmol/L) | | | ≥13.7 g/dL (i.e. ≥8.5 mmol/L) | | |
| Female | <12.1 g/dL (i.e. <7.5 mmol/L) | | | ≥12.1 g/dL (i.e. ≥7.5 mmol/L) | | |
| Reticulocytes | abnormal | | | normal | | |
| All cases | ≥2.5% of red blood cells | | | <2.5% of red blood cells | | |
| Creatinine | low | | normal | | high | |
| Male | <59 µmol/L | | 59 – 104 µmol/L | | >104 µmol/L | |
| Female | <45 µmol/L | | 45 – 84 µmol/L | | >84 µmol/L | |
| eGFR | abnormal | | | normal | | |
| All cases | ≤60 mL/min/1,73m^2^ | | | >60 mL/min/1,73m^2^ | | |
| LDH** | abnormal | | | normal | | |
| All cases | ≥450 E/L | | | <450 E/L | | |
| Serum iron | abnormal | | | normal | | |
| Male | <14 µmol/L | | | ≥14 µmol/L | | |
| Female | <10 µmol/L | | | ≥10 µmol/L | | |
| Folic acid | abnormal | | | normal | | |
| All cases | ≤5 nmol/L | | | >5 nmol/L | | |
| Vitamin B12 | abnormal | | | normal | | |
| All cases | <130 pmol/L | | | ≥130 pmol/L | | |
| Tests with categorized results | | | | | | |
| Ferritin | low | low normal | | high normal | | high |
| Male | <25 µg/L | 25 – 100 µg/L | | 100 – 250 µg/L | | >250 µg/L |
| Female | <20 µg/L | 20 – 100 µg/L | | 100 – 150 µg/L | | >150 µg/L |
| Leukocytes | abnormal | | | normal | | |
| All cases | <4.3 x 10^9^/L or >10 x 10^9^/L | | | 4.3 – 10 x 10^9^/L | | |
| Thrombocytes | abnormal | | | normal | | |
| All cases | <150 x 10^9^/L or >400 x 10^9^/L | | | 150 – 400 x 10^9^/L | | |
| MCV | low | | normal | | high | |
| All cases | <80 fL | | 80 – 100 fL | | >100 fL | |
| Transferrin | low | | normal | | high | |
| All cases | <2.0 g/L | | 2.0 – 3.6 g/L | | >3.6 g/L | |
| * = in 22 patients (10%) an alternative reference value was used – in male: 20 mm/h instead of 25 or 35 mm/h – in female: 30 mm/h instead of 35 mm/h  ** = in 29 patients (14%) an alternative reference value was used – 250 E/L instead of 450 E/L | | | | | | |

*eGFR = estimated glomerular filtration rate, ESR = erythrocyte sedimentation rate, LDH = lactate dehydrogenase, MCV = mean corpuscular volume.*

1. **Description of the multinomial logistic regression model**

For each of the 15 laboratory tests, the impact of each individual test (*within* the complete set of tests) on the ability of the GP to diagnose an underlying cause of anaemia (regardless whether or not correct) was assessed by fitting a multinomial logistic regression model (MLR) with a logistic link function to the data, using the mlogit-package and nnet-package [28, 29]. In this model, the diagnosis by the GP was set as dependent variable with five non-ordered categories (i.e. ACD, IDA, renal anaemia and other). The group with an ‘unknown’ cause was used as a reference. The 15 laboratory test results, as well as the age and gender of the patients served as predictors in each of the models. For the MCV, leukocytes, thrombocytes and transferrin test, a test result within the cut-off values was set as reference category. For ferritin, the ‘low normal’ test result (Table 1 of the manuscript) was set as reference. By fitting the MLR to the data, the regression coefficients and their corresponding significance level (p-value) were estimated for each of the predictors through maximum likelihood estimation. Regression coefficients estimated for the laboratory test show the relationship between the results of these tests and the *log odds* of choosing either IDA, ACD, renal anaemia or ‘other’ over the diagnosis ‘unknown’ (i.e. the reference category). Thereby, the regression coefficients of each predictor indicate how a one unit change in the test result influences the *log odds* of the GP diagnosing each of the underlying causes over ‘unknown’ when all other predictors stay the same. The resulting coefficients were exponentiated to improve their interpretability and evaluated for their significance.

1. **Description of the binomial logistic regression model**

The added value of each test on the probability of the GP diagnosing the *correct* underlying cause of anaemia was assessed by fitting a binomial logistic regression model (BLR) to the data. The dependent variable was the correctness of the diagnosis of the GP, i.e. a binary variable with categories ‘correct’ and ‘incorrect’. The ‘incorrect’ category was the reference category. The predictors were equal to the MLR as described earlier. As in the MLR, the regression coefficients and their corresponding significance level were estimated for each of the predictors by fitting the model to the data. Afterwards, the resulting regression coefficients were exponentiated and evaluated for their significance. In contrast to the MLR, the BLR shows the impact of a one unit change in the test result impacts the *(log) odds* of the GP diagnosing the *correct* underlying cause of anaemia over an *incorrect* underlying cause.

1. **Assumption testing**

Multicollinearity between the predictors of the selected most efficient subset models was assessed by determining the variance inflation factor (VIF) of each predictor. A VIF larger than five indicated a problematic level of multicollinearity, influencing the estimation of the regression coefficients when fitting the model [32]. Perfect separation of predictors by the outcome was evaluated while fitting each model as the coefficient estimates of the logistic regression models would not converge to finite values if this condition was present [33]. More specifically, it was evaluated whether (a certain range of values of) a predictor was associated with only one of the outcome values (i.e. one of the underlying causes of anaemia). A Hausman-McFadden diagnostic test was performed to determine whether the independence of irrelevant alternatives (IIA) assumption held [34], indicating that the choice to diagnose a specific underlying cause of anaemia should not depend on the presence or absence of a third underlying cause.

1. **Results of multinomial logistic regression model**

**Table S2** Selection of the best subset of laboratory tests for diagnosing an underlying cause of anaemia. Per iteration of the selection process, the table shows: the number of predictors included in the subset model, the Akaike Information Criterion (AIC) of the most efficient subset model and the predictor eliminated compared to the previous iteration.

| Selection Process | | |
| --- | --- | --- |
| Subset Size | AIC Model | Predictor Eliminated |
| 17 | 814.39 | - |
| 16 | 801.83 | Transferrin |
| 15 | 790.15 | MCV |
| 14 | 783.84 | Thrombocytes |
| 13 | 779.12 | Creatinine |
| 12 | 775.40 | Vitamin B12 |
| 11 | 772.42 | LDH |
| 10 | 770.93 | Gender |
| Most Efficient Subset | | |
| Subset Size | AIC Model | Resulting Predictors |
| 10 | 770.93 | Ferritin  CRP  Reticulocytes  Serum Iron  ESR  eGFR  Haemoglobin  Leukocytes  Folic acid  Age |

*CRP = C-reactive protein, eGFR = estimated glomerular filtration rate, ESR = erythrocyte sedimentation rate, LDH = lactate dehydrogenase, MCV = mean corpuscular volume.*

1. **Results of best subset selection**

**Table S3** Selection of the best subset of laboratory tests for diagnosing the correct underlying cause of anaemia. Per iteration of the selection process, the table shows: the number of predictors included in the subset model, the AIC of the most efficient subset model and the predictor eliminated compared to the previous iteration.

| Selection Process | | |
| --- | --- | --- |
| Subset Size | AIC Model | Predictor Eliminated |
| 17 | 497.65 | - |
| 16 | 495.70 | Gender |
| 15 | 493.82 | Thrombocytes |
| 14 | 492.05 | ESR |
| 13 | 490.32 | Leukocytes |
| 12 | 488.67 | Reticulocytes |
| 11 | 487.24 | Vitamin B12 |
| 10 | 485.88 | LDH |
| 9 | 484.71 | Haemoglobin |
| 8 | 483.25 | Serum Iron |
| 7 | 482.47 | Creatinine |
| 6 | 481.86 | eGFR |
| Most Efficient Subset | | |
| Subset Size | AIC Model | Resulting Predictors |
| 6 | 481.86 | Ferritin  CRP  MCV  Transferrin  Folic acid  Age |

*AIC = Akaike Information Criterion, CRP = C-reactive protein, eGFR = estimated glomerular filtration rate, ESR = erythrocyte sedimentation rate, LDH = lactate dehydrogenase, MCV = mean corpuscular volume.*

1. **Results of determining the variance inflation factor**

**Table S4** Variance inflation factors determined for assessing multicollinearity.

| Variance Inflation Factor – Resulting subset for diagnosing an underlying cause of anaemia | |
| --- | --- |
| Folic acid | 1.14 |
| Leukocytes | 1.16 |
| Reticulocytes | 1.21 |
| eGFR | 1.30 |
| Age | 1.34 |
| Haemoglobin | 1.66 |
| Ferritin | 1.96 |
| Serum Iron | 2.19 |
| CRP | 2.44 |
| ESR | 2.58 |
| Variance Inflation Factor – Resulting subset for diagnosing the correct underlying cause of anaemia | |
| Folic acid | 1.03 |
| Age | 1.14 |
| CRP | 1.46 |
| MCV | 2.07 |
| Ferritin | 2.66 |
| Transferrin | 2.99 |

*CRP = C-reactive protein, eGFR = estimated glomerular filtration rate, ESR = erythrocyte sedimentation rate, MCV = mean corpuscular volume.*
